# An improved *Bacillus subtilis* cell factory for producing *scyllo*-inositol, a promising therapeutic agent for Alzheimer’s disease

**DOI:** 10.1186/1475-2859-12-124

**Published:** 2013-12-11

**Authors:** Kosei Tanaka, Shintaro Tajima, Shinji Takenaka, Ken-ichi Yoshida

**Affiliations:** 1 Organization of Advanced Science and Technology, Kobe University, 1-1 Rokkodai, Nada, Kobe 657 8501, Japan; 2 Department of Agrobioscience, Graduate School of Agricultural Science, Kobe University, 1-1 Rokkodai, Kobe, Nada 657 8501, Japan

**Keywords:** *Bacillus subtilis*, *scyllo*-inositol, *myo*-inositol, Bioconversion, Alzheimer’s disease

## Abstract

**Background:**

*Bacillus subtilis* 168 possesses an efficient pathway to metabolize some of the stereoisomers of inositol, including *myo*-inositol (MI) and *scyllo*-inositol (SI). Previously we reported a prototype of a *B. subtilis* cell factory with modified inositol metabolism that converts MI into SI in the culture medium. However, it wasted half of initial 1.0% (w/v) MI, and the conversion was limited to produce only 0.4% (w/v) SI. To achieve a more efficient SI production, we attempted additional modifications.

**Results:**

All “useless” genes involved in MI and SI metabolism were deleted. Although no elevation in SI production was observed in the deletion strain, it did result in no wastage of MI anymore. Thus additionally, overexpression of the key enzymes, IolG and IolW, was appended to demonstrate that simultaneous overexpression of them enabled complete conversion of all MI into SI.

**Conclusions:**

The *B. subtilis* cell factory was improved to yield an SI production rate of 10 g/L/48 h at least. The improved conversion was achieved only in the presence of enriched nutrition in the form of 2% (w/v) Bacto soytone in the medium, which may be due to the increasing demand for regeneration of cofactors.

## Background

**Figure 1:**
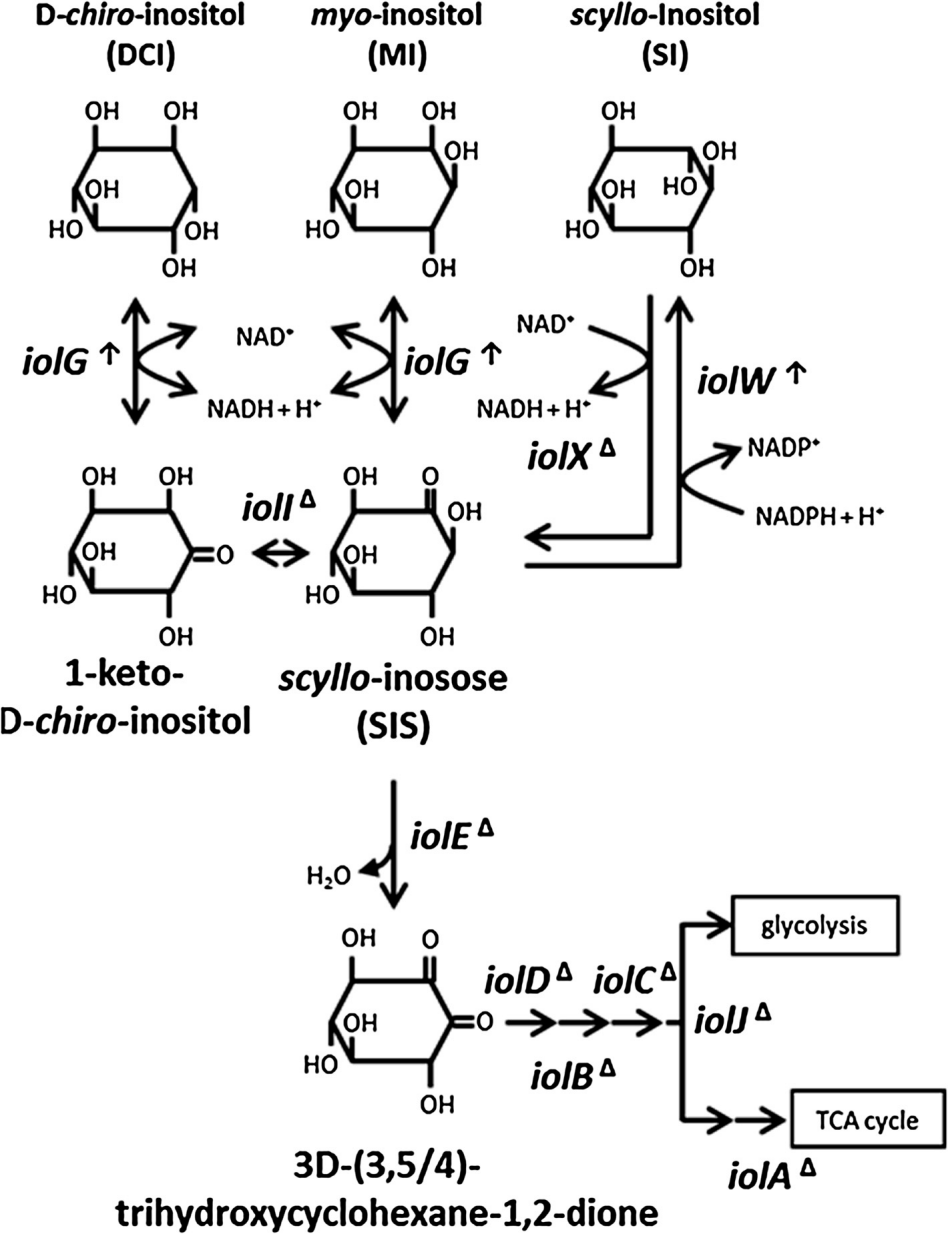
**Inositol metabolism in *B*. *subtilis.****B. subtilis iol* genes encoding enzymes for reactions in the inositol catabolic pathway and the respective intermediate compounds are shown. The symbols **↑** and Δ indicate over-expressed and deleted genes in KU106, respectively.

Among the 9 inositol (1,2,3,4,5,6-cyclohexanehexol) stereoisomers, *myo*-inositol (MI) is the most abundant in nature (Figure 1). It serves as an indispensable structural basis for a number of secondary messengers in eukaryotic cells [1]. In contrast, the other inositol stereoisomers are relatively rare, but some are known to exert specific health-promoting effects. In particular, *scyllo*-inositol (SI) has been regarded as a promising therapeutic agent for Alzheimer’s disease [2], one of the most common and problematic forms of dementia. Amyloid-beta (Aβ) aggregation and amyloid formation in the brain are key pathological features of Alzheimer’s disease [3]. SI directly interacts with the Aβ peptide and blocks the development of its fibrous aggregation [4]. In fact, oral administration of SI to a mouse model of Alzheimer’s disease inhibited Aβ aggregation, attenuated Aβ-induced impairments of spatial memory, reduced cerebral Aβ pathology, and decreased the rate of mortality [2]. Therefore, SI has received a fast-track designation from the US Food and Drug Administration for treatment of mild to moderate Alzheimer’s disease.

*Bacillus subtilis* is one of the best-studied Gram-positive bacteria and has the ability to metabolize at least three inositol stereoisomers including MI, SI, and D-*chiro*-inositol (Figure 1) [5]. The *B. subtilis iolABCDEFGHIJ* operon encodes enzymes involved in multiple steps of inositol metabolism, and transcription of the operon is regulated by the IolR transcriptional repressor, whose gene is located immediately upstream of the operon with divergent orientation [6]. In the first step, MI is converted to *scyllo*-inosose by the MI dehydrogenase IolG, with NAD^+^ reduction. IolG also dehydrogenates D-*chiro*-inositol to 1-keto-D-*chiro*-inositol, which is subsequently isomerized by IolI to *scyllo*-inosose [7]. In the second step, *scyllo*-inosose is dehydrated by IolE to 3D-(3,5/4)-trihydroxycyclohexane-1,2-dione [8]. This intermediate is metabolized sequentially by IolD, IolB, IolC, IolJ, and IolA, resulting in intermediates that enter glycolysis and the TCA cycle as dihydroxyacetone phosphate and acetyl-CoA, respectively [9].

*B. subtilis* possesses two additional and distinct inositol dehydrogenases, IolX and IolW, which act specifically on SI with NAD^+^ and NADP^+^ reduction, respectively [10]. Each of these enzymes can convert SI to *scyllo*-inosose, and *scyllo*-inosose is readily degraded further via the metabolic pathway described above [10]. Inactivation of *iolX* severely impairs cell growth depending on SI as the carbon source, whereas inactivation of *iolW* does not alter cell growth at all [10]. These results suggest that IolX can play the major physiological role in SI catabolism, whereas IolW may function through other mechanisms, such as reduction of *scyllo*-inosose into SI with oxidization of NADPH, as demonstrated *in vitro*[10].

In a previous study, we modified the metabolism of inositol to construct strains that could convert MI into SI. Strain TM039, a prototype of a cell factory, achieved the maximum rate to convert nearly half of initial MI into SI after 72 h of cultivation [5]. In this strain, three genes, including *iolR*, *iolX,* and *iolI*, were disrupted and a missense mutation *iolE41* was introduced. Those modifications were designed to enable constitutive expression of the *iolABCDEFGHIJ* operon, including *iolG*, and to disable dehydrogenation of SI as well as isomerization and dehydration of *scyllo*-inosose. Thus, MI was readily dehydrogenated to *scyllo*-inosose, which was accumulated and converted into SI by IolW, with the resulting SI appearing in the medium. However, this strain was found to consume and waste almost half of the MI initially contained in the medium [5].

In this study, we eliminated not only *iolE* but also all the other *iol* genes required for the latter steps in the metabolic pathway. We then overexpressed the two key enzymes for the conversion, IolG and IolW, to increase the conversion efficiency.

## Results and discussion

### Deletion of *iol* genes irrelevant to the conversion of MI into SI

Strain TM039, the prototype *B. subtilis* cell factory for producing SI, is able to convert MI into SI [5]. However, it appeared to waste half of the MI in the medium, given that only 0.4% (w/v) SI remained after the conversion from the initial 1.0% (w/v) MI (Table 1). We speculated that this loss was due to the residual activity of *scyllo*-inosose dehydratase encoded by *iolE41*[8]. Because the *iolE41* allele could not support growth on MI as a sole carbon source, we supposed that the IolE41 enzyme was severely impaired. However, it was still possible that the remaining limited activity of the mutated enzyme was involved in the wastage of MI. With the aim of eliminating the possibility, not only *iolE* but also *iolABCDFHIJ*, *iolX*, and *iolR* were deleted from the chromosome using a marker-free deletion technique [11] to yield strain MYI04. As expected, MYI04 did not waste MI after 48 h of cultivation (Table 1), although the deletion did not elevate the concentration of SI. We then speculated that the limited conversion of MI into SI resulted not from MI wastage but from the limited conversion capacity.

**Table 1:** MI and SI contained in the culture media after bioconversion

**Strain**	**MI concentration [% (w/v)]**^**a**^	**SI concentration [% (w/v)]**^**a**^
TM039	ND^b^	0.40
MYI04	0.59	0.41
KU101	0.53	0.47
KU102	0.56	0.44
KU104	ND^b^	0.74
	(ND^b,c^)	(0.85^c^)
KU105	0.13	0.80
KU106	ND^b^	1.00

^a^The medium initially contained 1.0% (w/v) MI. After 48 h of bioconversion, concentrations of MI and SI were determined as described in the text. Values are representative data from 3 independent measurements with similar results.

^b^ND, not detected (<0.001%).

^c^Concentrations of MI and SI at 24 h of bioconversion.

### Overexpression of *iolG* and *iolW*

In addition to the above-mentioned deletion, we next attempted to overexpress the two genes for the key enzymes IolG and IolW with the aim of enhancing the two reactions involved in the conversion (Figure 1).

**Figure 2:**
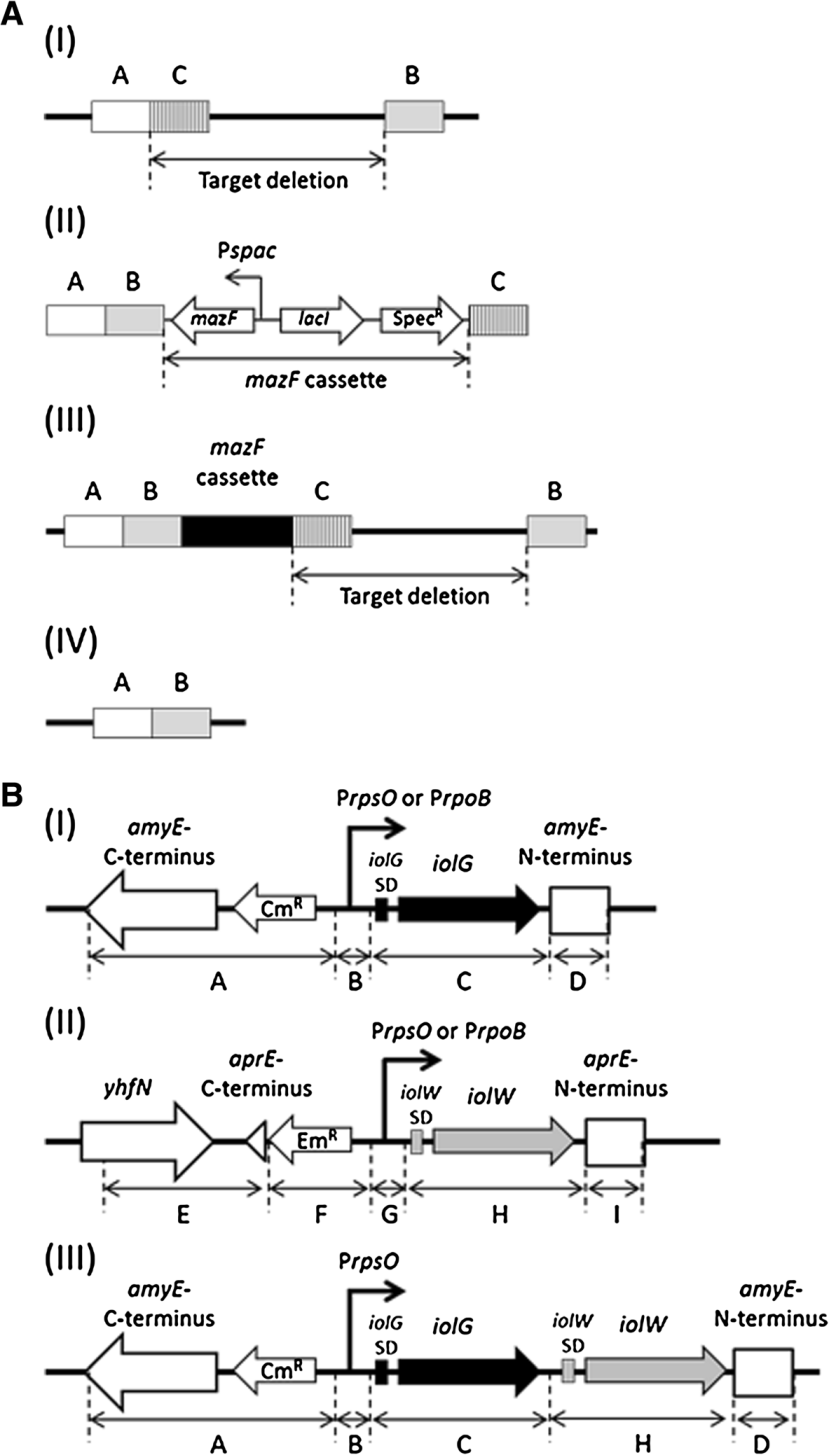
**Construction of marker-free deletion mutants and overexpression strains of *iolG *and *iolW*. (A)** Outline of the method for construction of marker-free deletion mutants. (A-I) Positional relationship between target deletion and regions A, B, and C contained in the PCR fragments used for construction of a marker-free deletion. A: upstream sequence; B: downstream sequence; C: sequence for integration of the *mazF* cassette. (A-II) Recombinant PCR product using fragments A, B, C, and the *mazF* cassette. (A-III) Integrant of the *mazF* cassette at the target region via a double crossover at regions A and C. An intrachromosomal single crossover event between the 2 directly repeated regions B results in elimination of the *mazF* cassette as well as the target region. (A-IV) Final structure of marker-free deletion. **(B)** Organization of the *iolG* and *iolW* overexpression cassettes. (B-I) For *iolG* overexpression, PCR fragments covering regions A + B (containing P*rpsO* or P*rpoB*) + C + D were ligated by recombinant PCR and integrated into the *amyE* locus by a double crossover event. (B-II) For *iolW* overexpression, PCR fragments covering regions E + F + G (P*rpsO* or P*rpoB*) + H + I were ligated and integrated into the *aprE* locus for construction of an *iolW-*overexpressing strain. (B-III) For simultaneous overexpression of *iolG* and *iolW*, a PCR fragment covering regions A + B + C + H + D was ligated and integrated into the *amyE* locus.

Global transcriptome analysis of *B. subtilis* grown under 104 different growth conditions allowed us to evaluate the strength and functional conditions of 2935 transcriptional promoters identified or predicted to date [12]. Among this large number of promoters, we chose two promoters of *rpsO* and *rpoB*, referred to as P*rpsO* and P*rpoB*, respectively, for overexpression of *iolG*, judging them to be the strongest and constitutively active even during stationary growth (Figure 2B). Strains KU101 and KU102 were thus constructed, in which *iolG* was integrated into the *amyE* locus and expressed under the control of P*rpsO* and P*rpoB* (P*rpsO-iolG* and P*rpoB-iolG,* respectively). Both strains exhibited conversion rates almost equal to that of MYI04 (Table 1), indicating that increasing expression of *iolG* alone was not sufficient to improve the conversion.

**Figure 3:**
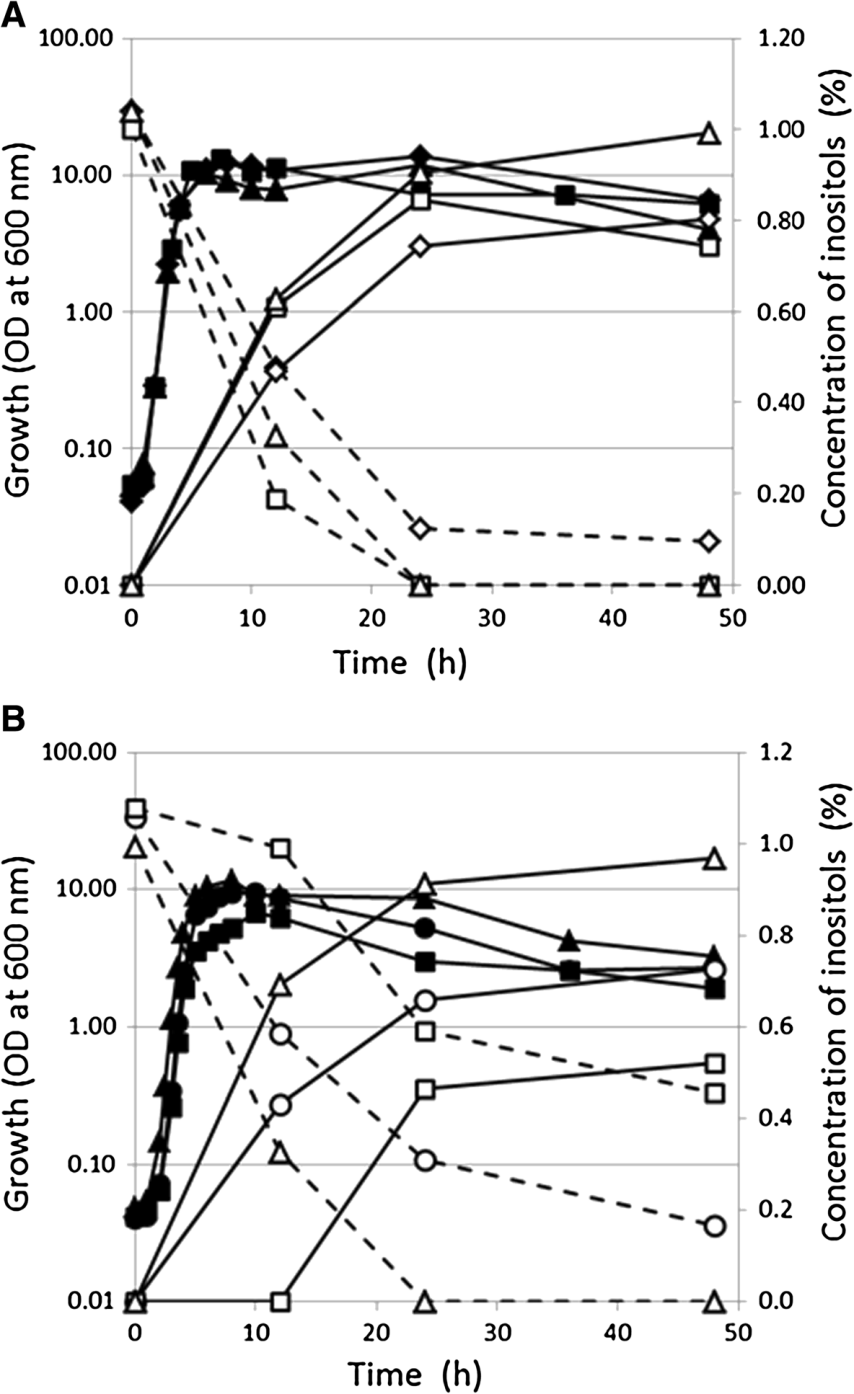
**Time course of SI production and MI consumption along with cell growth. (A)** Bioconversion from MI into SI was performed by strains KU104 (P*rpsO-iolW;* squares), KU105 (P*rpoB-iolW;* diamonds), and KU106 (P*rpsO*-*iolG iolW;* triangles). Cell growth (optical density of cells: closed symbols) and concentrations of MI (open symbols with dotted lines) and SI (open symbols with continuous lines) in the medium are shown. **(B)** Bioconversion of MI into SI was performed by strain KU106 (P*rpsO-iolG iolW*) in the presence of various concentrations of Bacto soytone; 2% (w/v) (triangles), 1% (circles), and 0.5% (squares). Cell growth (optical density of cells: closed symbols) and concentrations of MI (open symbols with dotted lines) and SI (open symbols with continuous lines) in the medium are shown. A representative set of data from three independent experiments with similar results is shown.

Next, overexpression of *iolW* was tested similarly. Overexpression of *iolW* markedly elevated the conversion of MI into SI; concentrations of SI reached up to 0.85% (w/v) at 24 h in strain KU104 (P*rpsO-iolW*) and 0.80% (w/v) at 48 h in KU105 (P*rpoB-iolW*) cultures (Table 1 and Figure 3A). These results suggested that intracellular levels of the IolW enzyme could be one of the important determinants in the conversion of MI into SI and also that P*rpsO* performed slightly better than P*rpoB* in overexpressing *iolW*. However, even the increased supply of IolW enzyme did not result in the conversion of all MI into SI. Accordingly, the additional strain KU106, which simultaneously overexpressed *iolG* and *iolW* under the control of P*rpsO*, was constructed. KU106 achieved the ultimate conversion to yield 1.0% (w/v) SI, equal to the initial concentration of MI (Figure 3B), representing the best SI production rate of 10 g/L/48 h at least. These results indicate that the synchronized acceleration of the two reactions enabled by excess IolG and IolW was required for the ultimate conversion.

### Culture conditions enabling efficient conversion

In a previous study, when the major nutrient, 2% (w/v) Bacto soytone, contained in the conversion medium was reduced by half, no severe effect on cell growth was observed, whereas SI production was impaired significantly [5]. We conducted a similar test on the conversion performed by KU106. As shown in Figure 3B, when the amount of Bacto soytone in the medium was reduced by one half and one quarter, SI production was reduced to 0.72% (w/v) and 0.52% (w/v), respectively. Given that the two reactions in the conversion catalyzed by IolG and IolW require reduction and oxidation of the cofactors NAD^+^ and NADPH, respectively (Figure 1), the results suggest that higher concentrations of Bacto soytone might be involved in the regeneration of NAD^+^ and NADPH. Assuming there could be some mechanism for cofactor regeneration, we are currently conducting an intensive investigation.

*B. subtilis* possesses two distinct inositol transporters, IolT and IolF, which differ in substrate specificity to MI and D-*chiro*-inositol [13,14]. In this study, we deleted *iolF* to construct MYI04, and the results indicated that *iolF* does not influence SI production and that *iolT* alone was enough for the ultimate conversion. However, we are still unable to explain how SI was secreted into the growth medium and thus, plan to identify the as-yet unknown efflux pump.

## Conclusions

We have described the second generation of a *B. subtilis* cell factory that permits SI production at a rate of 10 g/L/48 h at least. The cell factory contains multiple deletions of many *iol* genes and simultaneous overexpression of *iolG* and *iolW* for the key reactions, enabling efficient conversion of MI to SI. The highest conversion efficiency was achieved only in the presence of enriched nutrition in the form of 2% (w/v) Bacto soytone in the medium.

## Methods

### Bacterial strains, culture conditions, and primers

Bacterial strains and oligonucleotide primers used in this study are listed in Tables 2 and 3, respectively. Bacterial strains were maintained in Luria–Bertani (LB) medium [15]. Antibiotics, including 0.5 μg/mL erythromycin, 100 μg/mL spectinomycin, and 5 μg/mL chloramphenicol, were added as required, and 1 mM IPTG was also added to the selection medium when needed. To perform the inositol bioconversion, 100 mL of bioconversion medium, consisting of 2% (w/v) Bacto soytone (Becton, Dickinson and Co., Sparks, MD), 0.5% (w/v) Bacto yeast extract (Becton, Dickinson and Co.), 0.2% (w/v) glucose, and 1% (w/v) MI, was added to a 500-mL flask with baffles, inoculated with strains of *B. subtilis* at an optical density of 0.05 at 600 nm, and incubated at 37°C with shaking at 200 rpm.

**Table 2:** Bacterial strains used in this study

**Strain**	**Relevant genotype**	**Source or reference**
168	*trpC2*	Laboratory stock
TM039	*iolE41 metC7 iolR*::*cat iolI*::*spc iolX*::pMutin4(*erm*)	[5]
MYI04	Δ*iolABCDEF* Δ*iolHIJ* Δ*iolX* Δ*iolR*	This study
KU101	*amyE*:: P*rpsO-iolG* (*cat*) (MYI04 background)	This study
KU102	*amyE*:: P*rpoB*-*iolG* (*cat*) (MYI04 background)	This study
KU104	*aprE*:: Pr*psO-iolW* (*erm*) (MYI04 background)	This study
KU105	*aprE*:: P*rpoB-iolW* (*erm*) (MYI04 background)	This study
KU106	*amyE*:: P*rpsO-iolG-iolW* (*cat*) (MYI04 background)	This study

**Table 3:** Oligonucleotide primers used in this study

**Name**	**Sequence (5′ → 3′)**
iolR-A-F	TGCGCTGCGTAATCAATATC
iolR-A-R	GGCTTTGTTGATATTGTACTTATAAAAAACTCCTTCTTGAAT
iolR-B-F	ATTCAAGAAGGAGTTTTTTATAAGTACAATATCAACAAAGCC
iolR-B-R	GCTTGAGTCAATTCCGCTGTCGTTGAATTCACGCAGCACTTC
iolR-C-F	ATTAACGTACTGATTGGGTAGGATCCGCGCTGATGCGGATTCAGGAAAT
iolR-C-R	AAGGAGCGGGTTTTTCTCTT
iolAF-A-F	GGCCAGATGAATGCCGATTT
iolAF-A-R	ACGCCAATACGTAAACTCATTCTTATTGCCTCCTTCATTA
iolAF-B-F	TAATGAAGGAGGCAATAAGAATGAGTTTACGTATTGGCGT
iolAF-B-R	GCTTGAGTCAATTCCGCTGTCGGCGTGTCGACTACAGCCATA
iolAF-C-F	ATTAACGTACTGATTGGGTAGGATCCGCGTCAGGATGTTGAAGGGGAAG
iolAF-C-R	AGTTTGCCAAGCGTCACTTT
iolHIJ-A-F	CATGAAATTGACGTGCTCCA
iolHIJ-A-R	CTCGGCGGTTTCTGGTCTCTTTAGTTTTGAACTGTTGTAA
iolHIJ-B-F	TTACAACAGTTCAAAACTAAAGAGACCAGAAACCGCCGAG
iolHIJ-B-R	GCTTGAGTCAATTCCGCTGTCGAAACGCAGTTCAAACCGTTC
iolHIJ-C-F	ATTAACGTACTGATTGGGTAGGATCCGCGCGCACTCGTTTTCTTCAACA
iolHIJ-C-R	AATGGCTTCCTCAGCAGTC
iolX-A-F	GCTCCGACTGCTATTTTTGC
iolX-A-R	TAAGCGCGCTTCACATCTAGCAATACTGCACATCTTACTT
iolX-B-F	AAGTAAGATGTGCAGTATTGCTAGATGTGAAGCGCGCTTA
iolX-B-R	GCTTGAGTCAATTCCGCTGTCGCGGAGGAAACTGCCTATCAA
iolX-C-F	ATTAACGTACTGATTGGGTAGGATCCGCGGCCTTGAGGAATCAAAAGCA
iolX-C-R	TCCGTATGGAGAGGTTCTGC
amyE-1-F	CCTTCCAGGGTATGTTTCTC
amyE-1-R	CAAACGAAAATTGGATAAAGTGGG
PrpsO-iolG-F	CCCACTTTATCCAATTTTCGTTTGATGGCATCAAAGAATTAACTGAGC
PrpsO-iolG-R	CCTTTCTTTACTTGGCTCTGAGGCCAAATCATATTTAGCCCCAGTTACC
PrpoB-iolG-F	CCCACTTTATCCAATTTTCGTTTGGCGCGCCTTCTGCCATTG
PrpoB-iolG-R	CCTTTCTTTACTTGGCTCTGAGGGCGTATTATATGTGTAATAAGCATTTC
iolG-F	CCTCAGAGCCAAGTAAAGAAAGG
iolG-R2	CACAAATTAAAAACTGGTCTGATCGCCTCTGTTTTTTAGTTTTGAACTGTTG
amyE-2-F	CGATCAGACCAGTTTTTAATTTGTG
amyE-2-R	TTAACAAAATTCTCCAGTCTTCACATCG
aprE-1-F	CAATCTTTACGCTTTGCGTTCTCG
aprE-1-R	GTTACACGTTACTAAAGGGAATGTAGCGGAGCAGCAGCGTTAATTC
Em-F	CTACATTCCCTTTAGTAACGTGTAAC
Em-R	GAGTGTGTTGATAGTGCAGTATC
PrpsO-iolW-F	GATACTGCACTATCAACACACTCATGGCATCAAAGAATTAACTGAGC
PrpsO-iolW-R	GTATATACCCTCCTGATCAAATGGCCAAATCATATTTAGCCCCAGTTACC
PrpoB-iolW-F	GATACTGCACTATCAACACACTCGCGCGCCTTCTGCCATTG
PrpoB-iolW-R	GTATATACCCTCCTGATCAAATGGGCGTATTATATGTGTAATAAGCATTTC
iolW-F	CCATTTGATCAGGAGGGTATATAC
iolW-R	GATTGCGCGTGCGAAAGAAG
aprE-2-F	CTTCTTTCGCACGCGCAATCCGCAAACAACAAGCTGATCCAC
aprE-2-R	GACATTCGGCACACTCCTTTTC
iolG-R	CCTCTGTTTTTTAGTTTTGAACTGTTG
iolW-F-Tail	CAACAGTTCAAAACTAAAAAACAGAGGCCATTTGATCAGGAGGGTATATAC
iolW-R-Tail	CGATGTGAAGACTGGAGAATTTTGTTAAGATTGCGCGTGCGAAAGAAG
mazFK7-F	CGACAGCGGAATTGACTCAAGC
mazFK7-R	CGCGGATCCTACCCAATCAG

### Mutant construction

MYI04 (*ΔiolR, ΔiolABCDEF, ΔiolHIJ, ΔiolX*) was constructed by employing the marker-free deletion technique [11]. First, approximately 500-bp fragments located upstream (region A as indicated in Figure 2A-I) and downstream (region B) of the target deletion were amplified by PCR. Next, an additional fragment (region C) located immediately downstream of fragment A was amplified. These three fragments and the *mazF* cassette, consisting of *mazF* for suicidal toxin under the control of IPTG-inducible promoter (P*spac*), *lacI* for Lac repressor controlling P*spac*, and the spectinomycin resistance gene, were ligated by recombinant PCR to generate a long fragment containing the stretches corresponding to the regions A, B, the *mazF* cassette, and C in that order, as shown in Figure 2A-II. The recombinant PCR long fragments were used to transform the parental *B. subtilis* strain to be spectinomycin resistant via a double crossover in the homologous regions A and C, and thus the *mazF* cassette was introduced into the targeted region (Figure 2A-III). The spectinomycin-resistant transformants were then screened on IPTG-containing plates for detection of spectinomycin-sensitive mutants. In such mutants, an intrachromosomal single crossover event between the two direct repeat stretches corresponding to region B occurred to eliminate the *mazF* cassette and resulted in marker-free deletion between regions A and B (Figure 2A-IV). To construct *iolR* deletion, fragments of regions A, B, and C were amplified using iolR-A-F/iolR-A-R, iolR-B-F/iolR-B-R, and iolR-C-F/iolR-C-R primer pairs (Table 3), respectively. For *iolABCDEF* deletion, the primer pairs iolAF-A-F/iolAF-A-R, iolAF-B-F/iolAF-B-R, and iolAF-C-F/iolAF-C-R, respectively, were used. Similarly, for *iolHIJ* deletion, iolHIJ-A-F/iolHIJ-A-R, iolHIJ-B-F/iolHIJ-B-R, and iolHIJ-C-F/iolHIJ-C-R, respectively, were used, whereas for *iolX* deletion, iolX-A-F/iolX-A-R, iolX-B-F/iolX-B-R, and iolX-C-F/iolX-C-R, respectively, were used.

Strains KU104 and KU105 overexpressing *iolG* were constructed as follows. Figure 2B-I shows the schematic organization of the gene cassettes for overexpressing *iolG*. DNA fragments corresponding to regions A, B, C, and D were the elements of the cassette prepared by PCR. The DNA fragment for region A was amplified from the pCRE-test plasmid [16] by PCR using the primer pair amyE-1-F/amyE-1-R, and those for regions C and D were amplified from the chromosomal DNA of strain 168 using the pairs iolG-F/iolG-R2 and amyE-2-F/amyE-2-R, respectively. For region B, two distinct fragments containing P*rpsO* and P*rpoB* (referred to as B1 and B2, respectively) were amplified from 168 DNA by PCR using PrpsO-iolG-F/PrpsO-iolG-R and PrpoB-iolG-F/PrpoB-iolG-R, respectively. Two sets of the four fragments, covering the regions A + B1 + C + D and A + B2 + C + D, were ligated by recombinant PCR using the outside primers amyE-1-F and amyE-2-R. Each of the resulting two recombinant PCR products possessed *iolG* under the control of P*rpsO* or P*rpoB* flanked by N-terminal and C-terminal regions of *amyE* gene at either end (Figure 2B-I). Strain MYI04 was transformed to be chloramphenicol resistant by a double crossover recombination at the *amyE* locus to yield KU101 and KU102 as described [16].

Similarly, strains KU104 and KU105 overexpressing *iolW* were constructed as follows. Figure 2B-II shows the gene cassette for iolW overexpression. DNA fragments for the regions E, F, H, and I were amplified by PCR using the primer pairs aprE-1-F/aprE-1-R, Em-F/Em-R, iolW-F/iolW-R, and aprE-2-F/aprE-2-R, respectively. Two fragments for regions G containing P*rpsO* and P*rpoB* (referred to as G1 and G2, respectively) were amplified using PrpsO-iolW-F/PrpsO-iolW-R and PrpoB-iolW-F/PrpoB-iolW-R, respectively. Two sets of the five fragments, covering the regions E + F + G1 + H + I and E + F + G2 + H + I, were ligated by recombinant PCR with the outside primers of aprE-1-F and aprE-2-R and used to transform MYI04 by a double crossover recombination at the *aprE* locus to obtain KU104 and KU105, respectively.

Strain KU106, simultaneously overexpressing *iolG* and *iolW*, was constructed as follows. DNA fragments for regions A, B, and D were amplified by PCR as described above (Figure 2B-I). Fragments for regions C and H were amplified using the primer pairs iolG-F/iolG-R and iolW-F-tail/iolW-R-tail, respectively (Figure 2B-I and II). These five fragments, covering regions A + B + C + H + D, were ligated by recombinant PCR with outside primers of amyE-1-F and amyE-2-R. The resulting PCR product, containing *iolG* and *iolW* genes under the control of P*rpsO* flanked by the N-terminal and C-terminal regions of *amyE*, was used to transform MYI04 by a double crossover recombination at the *amyE* locus to obtain KU106.

### Measurement of MI and SI in medium

Bacterial culture was diluted appropriately with pure water when required, and cells were removed from the culture medium by centrifugation. The supernatant was mixed with AG 50 W-X8 resin (Bio-Rad, Hercules, CA) for 1 h at 4°C, and then passed through an Amicon Ultra-0.5 ml 3 K Centrifugal Filter (Millipore, Billerica, MA). The eluent was subjected to high-performance liquid chromatography (LaChrom Elite: HITACHI High Technologies, Tokyo, Japan) with refractive index detection using a Wakosil5NH2 column (4.6 × 250 mm) (Wako Pure Chemical Industries, Osaka, Japan) maintained at 25°C with a flow of acetonitrile/water (80/20) at 2 mL/min. The retention time was used to identify the stereoisomers, and refractive index units were used to calculate their concentrations.

## Abbreviations

MI: *myo*-inositol; SI: *scyllo*-inositol; Aβ: Amyloid-beta.

## Competing interests

The authors declare that they have no competing interests.

## Authors’ contributions

Conception and design of the study: KY. Acquisition of data: KT and STj. Analysis and interpretation of data: KT. Drafting the article: KT. Revising it critically for important intellectual content: KY and STk. Final approval of the version to be submitted: all co-authors. All authors read and approved the final manuscript.
